# Immunomodulatory agents and cell therapy for patients with type 1 diabetes

**DOI:** 10.20945/2359-4292-2024-0233

**Published:** 2025-03-24

**Authors:** Melanie Rodacki, Karina Ribeiro Silva, Debora Batista Araujo, Joana R. Dantas, Maria Eduarda Nascimento Ramos, Lenita Zajdenverg, Leandra Santos Baptista

**Affiliations:** 1 Departamento de Medicina Interna, Universidade Federal do Rio de Janeiro, Rio de Janeiro, RJ, Brasil; 2 Laboratório de Pesquisa com Células-Tronco, Departamento de Histologia e Embriologia, Instituto de Biologia, Universidade do Estado do Rio de Janeiro, Rio de Janeiro, RJ, Brasil; 3 Novo Nordisk Pharmaceutics, Denmark, Copenhagen, Zealand, Dinamarca; 4 NUMPEX-BIO, Campus Duque de Caxias Professor Geraldo Cidade, Universidade Federal do Rio de Janeiro, Rio de Janeiro, RJ, Brasil

**Keywords:** Type 1 diabetes, autoimmune, cell therapy

## Abstract

Type 1 diabetes (TID) is a chronic disease caused by autoimmune destruction of pancreatic β-cells, that progresses in three stages: 1) stage 1: β-cell autoimmunity + normoglycemia; 2) stage 2: β-cell autoimmunity + mild dysglycemia; 3) stage 3: symptomatic disease + hyperglycemia. Interventions to prevent or cure T1D in the various stages of the disease have been pursued and may target the prevention of the destruction of β cells, regression of insulitis, preservation or recovery of β cells residual mass. Some therapies show promising results that might change the natural history and the approach to patients with T1D in the next few years. Teplizumab, a humanized monoclonal antibody that binds to CD3, was recently approved in the USA to delay Stage 3 T1D in individuals ≥ 8 years of age. Other non-cellular immunomodulatory therapies, both antigen-specific and non-specific, have shown interesting results either in patients with stage 2 or recent onset stage 3 T1D. Cell therapies such as non-myeloablative transplantation of autologous hematopoietic stem cells, mesenchymal stem cells, and tolerogenic dendritic cells have been also studied in these individuals, aiming immunomodulation. Stem cell-derived islet replacement therapy is promising for patients with long-standing T1D, especially with asymptomatic hypoglycemia not resolved by technology. This review aimed to provide updated information on the main immunomodulatory agents and cell therapy options for type 1 diabetes.

## INTRODUCTION

Type 1 diabetes (TID) is a chronic disease caused by T-cell mediated autoimmune destruction of pancreatic β cells. Patients with T1D require lifelong insulin treatment, which may cause hypoglycemia and interfere in quality of life. T1D progresses in three stages. In stage 1, there is pancreatic β cell autoimmunity with two or more islet autoantibodies and normoglycemia. Stage 2 is characterized by pancreatic β cell autoimmunity and asymptomatic dysglycemia. In stage 3, there is symptomatic disease and hyperglycemia that fulfill criteria for diabetes (^1^). Disease-modifying therapies to prevent or cure T1D in its various stages have been pursued. This review aimed to provide updated information on the main immunomodulatory agents and cell therapy options for type 1 diabetes.

## AUTOIMMUNITY AND TYPE 1 DIABETES

In T1D, there is an autoimmune attack to the pancreatic β cells. Both cellular and innate immunity play a role in this process. Central and peripheral tolerance defects contribute to the failure in self-tolerance (^2^).

The initial pancreatic lesion is insulitis, an inflammation in the pancreatic β cells. Multiple immune cell populations play a role in the process and are identified in the insular region, but T cells predominate and have an essential role in the development of islet autoimmunity and progression to stage 3 T1D. The specific contribution of each T cell subset as well as their interplay with other immune and islet cells are not yet fully understood. The destruction of pancreatic β cells depends on a combination of CD4+ and CD8+ T cells, but the mechanisms that trigger and lead to T cell infiltration to islet cells have not been clarified. The infiltrating T cells react to autoantigens in the pancreatic β-cells such as insulin epitopes, glutamic acid decarboxylase, insulinoma-associated protein 2, zinc transporter 8 and heat shock protein 60 (^3^).

A combination of immunological, genetic and environmental factors may activate autoreactive CD8+ T cells. Pro-inflammatory cytokines such as IL-12, IFN-γ, and TNF-α can promote activation and differentiation of autoreactive CD8+ T cells. Molecular mimicry may also be implicated, which means pathogens can express antigens that mimic host proteins or share epitopes with self-antigens. Consequently, the immune response against the pathogen can divert to attack self-antigens. Aberrant antigen presentation by dendritic cells or other antigen-presenting cells, oxidative stress and regulatory T cell (Treg) dysfunction may also promote the activation of autoreactive T cells (^3,4,5^). Activated autoreactive CD8+ T cells can lead to secretion of cytotoxic cytokines and directly attack pancreatic β-cells (^4,5^). Regulatory T cells (Tregs) are recruited to the pancreas and inhibit β-cell destruction. Although in T1D this mechanism is not successful in preventing disease progression, it may be explored for cellular and non-cellular medical interventions. Macrophages, dendritic cells (DC) and B lymphocytes are also found in the infiltrative pancreatic lesion (^4^).

Autoantibodies produced by B lymphocytes are observed in the serum during the development of T1D and can be used as biomarkers for T1D-related autoimmunity. The main antibodies are directed against insulin (IAA), glutamic acid decarboxylase 65 (GADA), Insulinoma associated protein 2 antigen (IA-2A) and zinc transporter 8 (ZnT8A). Antibodies seem to be predominantly a reflection of the lesion with massive antigen liberation rather than the cause of damage. B lymphocytes seem to participate in the pathogenesis of the disease, but probably as antigen-presenting cells (APCs) (^6,6,7,8^).

T1D development is a multifactor process, with the contribution of genetic and environmental factors. Genetic risk for T1D is conferred mostly by polymorphisms in the Human Leukocyte Antigen (HLA) complex, the human major histocompatibility complex (MHC), specifically with class II HLA (especially HLA DRB1*03:01-DQB1*02:01/DRB1*04 -DQ8 genotype). Class I HLA and more than 70 common non-HLA T1D risk loci are also involved, especially in insulin gene, *PTPN22* and *CTLA4*. Polygenic scores have been proposed to aggregate risk variants from associated loci into a single number, to estimate the genetic risk of T1D (^9,10,11^).

Infections, gut microbiota and nutrition are the main proposed environmental risk factors for T1D. Viral infections, especially mumps, rubella, enterovirus (such as Cocksakie B virus) and cytomegalovirus (CMV) infection may lead to local inflammation, which probably promotes CD8+ T cells activation, cytotoxic reaction, β cells epitopes presentation, insulitis and pancreatic β-cell destruction (12. The TEDDY study group has shown that children who later developed islet autoimmunity have different immune responses to enterovirus when compared to controls (^13,14^).

Curiously, changes in life habits in the past decades, such as improvement in hygiene, vaccination and use of antibiotics might have promoted a decrease in microorganism exposure, a reduction of microbiota development and also in the ability to train the immune system to tolerate self-targets, which increased the appearance of autoimmune diseases, such as T1D (^15^). This has been known as “Hygiene theory”.

Nutritional factors may also play an important role in the development of T1D. Although some epidemiological retrospective studies have suggested that breast-feeding may have a protective role in T1D risk, a causal and definite relationship has never been confirmed. Prospective studies failed to demonstrate any impact of breastfeeding duration in the risk of T1D (^16,17^).

Metabolic syndrome and obesity might also influence the development of T1D. Obesity is associated with progression to stage 3 T1D (^18^) and the Accelerator Hypothesis predicts earlier onset of T1D in individuals with higher body mass (^19^).

Vitamin D (VITD) and zinc intake may have an impact in the immune system. 25-OH-Vitamin D levels and polymorphisms in the vitamin D receptor (VDR) gene may have a combined role in the development of pancreatic autoimmunity in children at increased genetic risk for T1D (^20,21,22^). Furthermore, there is an increased incidence of T1D in winter months, which may be associated with lower sun exposure and lower VIT D levels (^23,24^). *VDR* gene polymorphisms were associated with the risk of T1D in some (^25,26^), but not all studies (^27^).

The autoimmune pancreatic destruction observed in T1D is clinically evident through the presence of serum diabetes-related autoantibodies and progressive dysglycemia. Numerous clinical studies have explored the evolution of this process in pediatric populations, but information on the progression in adults is less well-documented. Adults may exhibit a slower evolution of autoimmune destruction, which could have clinical implications for their response to disease-modifying therapies (^28^).

In this context, two different endotypes have been described for T1D: 1) T1D endotype 1 (T1DE1), which is more frequent in patients younger than 7 years, associated with HLA DR4-DQ8 and greater lymphocyte infiltration; 2) T1D endotype 2 (T1DE2), which is more common in individuals older than 13 years and is associated with HLA DR3-DQ2. Patients with T1DE1 tend to respond well to immunomodulatory agents, unlike those with T1DE2. Additionally, T1DE1 is more commonly associated with celiac disease, while T1DE2 is more prone to develop thyroid autoimmunity (^29^). Biomarkers that could identify each endotype and their potential response to medications are of great interest, to develop precision medicine-based intervention strategies. Higher rates of aberrant proinsulin processing, leading to an elevated proinsulin to C-peptide ratio, seem characteristic of TIDE1 (^29^). Similar markers with potential clinical applications should be further investigated.

## DISEASE-MODIFYING INTERVENTIONS FOR TYPE 1 DIABETES

### Non-cellular immunomodulatory therapies

**Figure 1:**
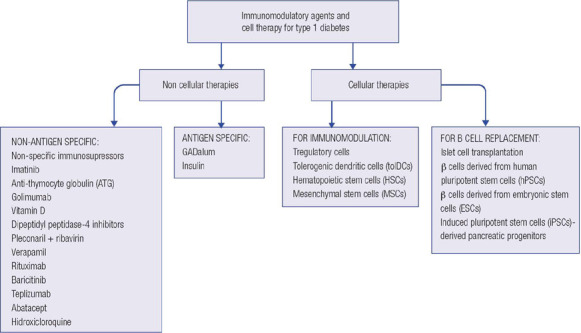
Disease-modifying therapies for type 1 diabetes.

Immunomodulation is a promising strategy to interrupt the autoimmune attack in T1D. The use of immunomodulatory agents as disease-modifying therapies for T1D represents a big challenge, as the treatment must provide an effective and long-lasting immunomodulation but with acceptable adverse effects. Antigen and non-antigen-based therapies have been investigated for recent-onset stage 3 T1D and, more recently, for stages 1 and 2. These interventions are summarized in Table 1 and Figure 1. This type of treatment is not suited for individuals with long-standing T1D, which already have significant destruction of β cells.

**Table 1:** Interventions that have been tested for T1D at each disease stage

Stage	Intervention
High genetic risk – no antibodies	• Hydrolyzed formula: No impact in the progression to T1D • Removal of bovine insulin from infant formula milk: No impact on the progression to T1D. • Delayed gluten exposure: No impact in the progression to T1D.
Pre-clinical stage	• Abatacept (stage 1): No impact in progression to stage 2 or 3. • Hydroxychloroquine (stage 1): No impact on progression to stage 2 or 3. • GAD-Alum: No impact on progression to stage 3. • Insulin (oral/subcutaneous): No impact on progression to stage 3. • Teplizumab: Delays the progression of stage 3 in individuals with stage 2 T1D.
Recent-onset stage 3*	NON-CELLULAR THERAPIES • Cyclosporine: Risks greater than temporary benefits. • Mycophenolate mofetil: No impact. • (alone or with daclizumab) • Imatinib: Higher C-peptide levels than conventional treatment. • Anti-thymocyte globulin (ATG): Higher C-peptide levels than conventional treatment. • Golimumab: Higher C-peptide and lower insulin requirement. • Rituximab: Higher C-peptide, lower insulin requirement, lower HbA1c • Baricitinib: Higher C-peptide and lower insulin requirement. • Teplizumab: Higher C-peptide, lower insulin requirement, lower HbA1c. • Abatacept: Higher C-peptide, lower insulin requirement, lower HbA1c. • Ustekimumab: Ongoing phase II trial. • Vitamin D: Conflicting results. Slightly higher C-peptide than conventional treatment in some studies. • Verapamil: Higher C-peptide than conventional treatment. • Pleconaril + ribavirin: Higher C-peptide than treatment solely with insulin. • Sitagliptin: For LADA, compared with insulin intervention alone, sitagliptin plus insulin appeared to maintain β-cell function and improve insulin sensitivity to some extent. • Microbiome-modulating agents: Higher C-peptide and lower HbA1c. • GAD-alum vaccination: Benefits in LADA and patients with HLD DR3/DQ2 CELLULAR THERAPIES • T regulatory cells: Phase I trials indicated higher C-peptide and lower insulin requirements. Phase II trials are ongoing. • Tolerogenic dendritic cells: Phase I and II trials ongoing. • Hematopoietic stem cells (HSC) therapy: Autologous nonmyeloablative HSC transplantation + high-dose cyclophosphamide plus ATG as a conditioning (immune ablative) regimen increased C-peptide and reduced insulin requirement. • Mesenchymal stem/stromal cells: Slightly lower insulin requirements, better glycemic control, and transient better pancreatic function but the potential benefits do not seem to be sustained.
Long-standing stage 3	• Islet cells transplantation: Transient insulin independence, requires immunosuppression; safe and well-suited for patients with brittle diabetes and severe hypoglycemia. • Stem-cell-derived differentiated β-like cells: Phase I/II trial with VX-880 cells has shown an increase in fasting and postprandial C-peptide, a decrease in HbA1c and reduced insulin requirement. Insulin independency was described in a few patients.

* In all trials, all participants received insulin.

#### Non-antigen-specific based therapies

Several therapies have been tested for stage 3 T1D aiming to reduce the loss of β cells and block the ongoing autoimmune attack.

Broad immunosuppression commonly used in transplantation provided evidence that immunotherapy can effectively preserve some pancreatic β cell function, but the risk profile is not acceptable for T1D. Cyclosporine is a calcineurin inhibitor of T cell receptor stimulation, which decreases T cell activation and interleukin 2 (IL-2) production. This drug has been associated with a better β cell function throughout the treatment period for patients with stage 3 T1D. However, glycemic control and C-peptide levels deteriorated after drug suspension. Since there was no long-lasting effect, risks were greater than benefits (^30,31^).

Mycophenolate mofetil is another non-specific immunosuppressor agent that has been evaluated for recent onset patients with stage 3 T1D. It inhibits T and B cells proliferation and has been tested alone or in association with Daclizumab, which is an anti-CD25 monoclonal antibody that inhibits IL-2 binding and progression of T lymphocytes through the cell cycle. Either agent alone or in combination did not improve pancreatic function after 2 years (^32^).

Newer selective immunotherapies that inhibit specific immune pathways relevant to the progression of T1D with few side effects have been tested, such as Imatinib, anti-thymocyte globulin (ATG) and Golimumab. Imatinib (Gleevec) is a tyrosine kinase that may impact both immunologic and metabolic pathways, reducing endoplasmic reticulum stress in pancreatic β-cells (reducing apoptosis), as well as improving insulin sensitivity in animal models. Newly diagnosed stage 3 T1D patients (n = 45) that received Imatinib were compared to placebo (n = 22). Those in the intervention arm had a higher C-peptide after 12 months, but this was not sustained after 2 years. HbA1c tended to be lower in the Imatinib patients in the first year, without a significant difference in insulin dose. Drug tolerance seemed to be better than seen in oncologic patients, but 13% had to permanently discontinue the medication (^33^).

Low-dose ATG has been tested in recent onset patients with stage 3 T1D. Pilot studies demonstrated that low-dose ATG + pegylated granulocyte colony stimulating factor (GCSF) achieved a relative increase of Tregs in circulation, but with no control arm. Haller and cols. investigated the use of ATG (2.5 mg/kg) + GCSF in 89 subjects with recent-onset stage 3 T1D (^29^) randomized to ATG + GCSF, 29 received ATG alone and 31 received placebo), in combination with insulin. After 12 months, patients that received ATG had a better pancreatic function than the placebo group, but this difference was not observed between the ATG + GCSF and placebo groups. HbA1c was lower in ATG or in ATG/GCSF groups than in the placebo group. Treatment was associated to a reduced in CD4/CD8 T cells ratio (^34^). A treatment protocol with different low dose ATG is on-going (2.5 mg/kg, 1.5 mg/kg, 0.5 mg/kg and 0.1 mg/kg ATG total dose) (^35^).

Golimumab is a human monoclonal antibody specific for tumor necrosis factor alpha. A phase 2, multicenter, placebo-controlled, double-blind trial investigated its use for recent onset subjects with stage 3 T1D (n = 84), during 52 weeks. Patients who received intervention showed higher C-peptide area under curve (AUC) than those treated with conventional therapy, with similar glycemic control, lower insulin requirement and without serious adverse events (^36^).

The role of B-lymphocyte depletion with rituximab, an anti-CD20 monoclonal antibody, has also been investigated in patients with recent-onset stage 3 T1D. In a phase 2 randomized double-blind study, 87 patients aged 8 to 40 years received infusions of rituximab or placebo. After 1 year, the mean AUC C-peptide was higher in the rituximab group than in the placebo group, with slightly lower levels of HbA1c and lower insulin requirement. More patients in the rituximab group than in the placebo group had adverse events, mostly after the first infusion, but they were generally mild. Therefore, B lymphocytes may contribute to the pathogenesis of T1D, which can be explored in future trials for the prevention or cure of T1D (^37^).

Baricitinib is a Janus kinase (JAK) inhibitor that blocks cytokine signaling and is an effective disease-modifying treatment for several autoimmune diseases, such as rheumatoid arthritis. In a phase 2, double-blind, randomized, placebo-controlled trial, patients with stage 3 T1D (n = 91) that received baricitinib (^4^) mg once per day) orally for 48 weeks had higher C-peptide, lower insulin requirement, similar HbA1c and lower glucose variation than those in placebo group. The frequency and severity of adverse events were similar in both groups, without serious adverse events (^38^).

Ustekinumab is a monoclonal antibody used in psoriasis to target interleukin 12 (IL-12) and 23 (IL-23) pathways. In a pilot open-label study with 20 adults with stage 3 T1D of recent-onset (<100 days), Marwaha e cols demonstrated that the intervention reduced the percentage of circulating T helper 17, T helper 1, and T helper 17.1 cells as well as proinsulin-specific T cells that secreted Interferon-γ and interleukin-17A (^39^). A double-blind phase II study to assess the safety and efficacy of Ustekinumab in children and adolescents aged 12 to 18 years with recent-onset stage 3 T1D is currently ongoing (^40^).

VIT D supplementation has also been investigated for T1D. The recent identification of VIT D receptors in several tissues not associated with calcium metabolism, such as pancreatic β cells, endothelium and immune system cells, has increased researchers’ interest in the systemic role of this prohormone (^41^). VIT D appears to act on both the adaptive and innate immune responses, modulating the maturation of dendritic cells (^42^). Furthermore, VIT D appears to play a suppressive role in the proliferation of lymphocytes, alter T helper1/T helper 2 response and act on Tregs. In vitro studies demonstrated that 1.25 OH vitamin D decreased apoptosis in human pancreatic β-cells by reducing the exposure of MHC class I molecules on the surface and increasing other anti-apoptotic molecules such as the A20 protein (^43^).

In EURODIAB multicenter study, VIT D supplementation in childhood was associated to a reduced risk of T1D, with an odds ratio of 0.67 (^44^). This modulation can even occur during intrauterine phase. VIT D supplementation in pregnant women is also associated with a lower risk of autoimmune diabetes in their offspring (^45^).

These data have led to intervention studies with VIT D in newly diagnosed T1D patients. A double-blind, placebo-controlled clinical trial carried out supplementation of 2,000 UI/day of cholecalciferol in 38 patients with newly diagnosed T1D. In the intervention group, there was a smaller reduction in C-peptide within 18 months and a trend towards an increase in Tregs (CD4+ CD25+ FOXP3+ cells) and interleukin 10 (^46^). Treiber and cols. demonstrated an enhanced inhibitory capacity of Tregs, reduced fasting glucose, HbA1c and insulin dose (^47^) in 30 patients with new onset stage 3 T1D assigned to cholecalciferol (^70^) IU/kg/day) *vs.* treatment with placebo for 12 months, in association with insulin. However, not all studies confirmed these benefits (^48^). These heterogeneous results may be secondary to the different doses and forms of VIT D supplementation (^49^).

Dipeptidyl peptidase-4 inhibitors (DPP-4i) might also have some effect in modulating inflammation and autoimmune β cell destruction. CD26 is a surface T cell activation antigen with DPP4 enzymatic activity that plays a central role in thymic maturation and costimulation, migration, and memory development of T cells (^50^). However, evidence to confirm this hypothesis is still lacking, as well as clinical information about the efficacy of DPP-4i as an immunomodulatory agent in stage 2 or 3 T1D. Yang and cols. have shown that patients with LADA (n = 51) who were treated with sitagliptin + insulin for 24 months appeared to have slightly better β-cell function and insulin sensitivity to some extent than those treated solely with insulin (^51^).

The use of antiviral treatment with pleconaril and ribavirin aiming to stop the autoimmune aggression has also been tested for recent-onset patients with stage 3 T1D. Previous studies showed a low-grade enterovirus infection in the pancreatic islets of patients with newly diagnosed T1D, which could lead to inflammation and pancreatic β-cell destruction. In Diabetes Virus Detection (DiViD), a phase 2, placebo-controlled, randomized double-blind trial, 96 children and adolescents with recent-onset stage 3 T1D received this intervention (n = 47) or placebo (n = 49) for 6 months. The treatment was well-tolerated and serum C-peptide AUC was higher in the intervention group compared to placebo at 12 months. This result indicates that antiviral treatment may preserve residual insulin production in children and adolescents with new-onset stage 3 T1D. HbA1c was lower in the intervention group than in the placebo group at 6 but not at 12 months, with similar insulin requirements (^52^).

Verapamil, a calcium channel blocker, has also shown interesting results for patients with stage 3 T1D. Preclinical studies showed that thioredoxin-interacting protein overexpression induced pancreatic β cell apoptosis and was involved in glucotoxicity-induced pancreatic β cell death, which could be reduced by calcium channel blockers. In a double-blind, randomized clinical trial including children and adolescents with new-onset stage 3 T1D, participants received once-daily oral verapamil (n = 47) or placebo (n = 41) during 52 weeks. Although groups had similar C-peptide levels at baseline, those in the intervention group had a 30% higher C-peptide level at 52 weeks, without differences in glycemic control and no serious adverse events (^53^).

Microbiome-Modulating Agents (MMAs) have also been tested for patients with T1D, as the gut microbiome has been linked to its pathogenesis. A systematic review and meta-analysis with high heterogeneity including 10 randomized controlled trials (*n* = 630 with variable disease duration) has shown that MMA supplementation is associated with improved fasting C-peptide and HbA1c. The study findings did not substantiate a favorable association between MMA intervention and C-reactive protein, TNF-α or IL-10. Further large-scale clinical trials are necessary to elucidate if MMAs have any influence in the progression of autoimmune pancreatic destruction (^54^). Lokesh and cols. investigated the use of probiotics supplementation in patients with new-onset T1D (n = 50) in a single-center randomized controlled clinical trial. Individuals who underwent intervention had a significant increase in the percentage of induced-T regulatory cells, IL-10 and C-peptide levels as well as a decrease in HbA1c (^55^).

Some immunomodulatory agents have been tested not only for stage 3 T1D but also for patients in other stages of T1D. Teplizumab is an Fc receptor-nonbinding anti-CD3 monoclonal antibody that has promising results for both stage 2 and stage 3 T1D. For those with recent-onset stage 3 T1D, a 14-day full-dose intervention induced less AUC C-peptide decline, lower insulin requirement, and better glycemic control than conventional treatment in a multicenter randomized trial (n = 516) after one year (^56^).

A decline in C-peptide response was seen afterward. Nevertheless, a reduction in the decline of β cell function may be seen for as long as seven years after the onset of stage 3 (^57,58,59^). Recently, a randomized placebo-controlled trial tested the impact of two 12-day courses (^26^) weeks apart) of intravenous teplizumab in patients with newly diagnosed stage 3 type 1 diabetes. At 78 weeks, patients treated with teplizumab (n = 217) had significantly higher stimulated C-peptide levels than those who received placebo (n = 111) (*P* < 0.001). Among patients treated with teplizumab, 94.9% maintained peak C-peptide level ≥ 0.2 pmol per milliliter, as compared to 79.2% of those receiving placebo (^60^), without significant differences in HbA1c and insulin dose.

For those in stage 2 T1D, teplizumab increased the median time to diagnosis of stage 3 T1D (48.4 *vs.* 24.4 months), with a hazard ratio for stage 3 T1D of 0.41. There were expected adverse events of rash and transient lymphopenia. Stage 3 T1D was diagnosed in 43% of patients who received teplizumab *vs.* 72% who received placebo (^61^). In an extended follow-up (923-day median), the median times to diagnosis were 59.6 and 27.1 months for the intervention and placebo groups, respectively (HR = 0.457, *P* = 0.01). Fifty percent of teplizumab-treated but only 22% of the placebo-treated remained diabetes-free. Teplizumab treatment improved β cell function and reversed a decline in insulin secretion before enrollment. The changes in C-peptide seen with the intervention were associated with increases in partially exhausted memory KLRG1+TIGIT+CD8+ T cells that showed reduced secretion of interferon γ and TNFα (^62^). This drug was approved by the FDA for clinical use in patients with stage 2 T1D in 2022, but still has limited availability and high cost. Long-term effects and safety profiles of teplizumab in children, adolescents and adults with early stages T1D are still lacking, but data from trials that included individuals with stage 3 T1D indicated that the safety profile seems acceptable, considering the potential benefits.

Abatacept is a selective modulator of CD80/86-CD28 costimulatory signal and inhibits T cell activation blocking specific binding of CD80/CD86 receptor in antigen-presenting cells to CD28 on T cells. It has been tested in different stages of T1D. In a multicenter, double-blind, randomized controlled trial, patients aged 6-45 years with recent-onset stage 3 T1D were assigned to receive abatacept (n = 77) or placebo (n = 35) infusions intravenously (^27^) infusions over 2 years). C-peptide AUC was 59% higher at 2 years with abatacept than with placebo. HbA1c levels were lower in the treatment group than the placebo group, but a baseline difference was also observed. Lower insulin requirement was observed at 6 and 12 months in the intervention group. The study reported few infusion-related adverse events, no increase in infections or neutropenia (^63^). The drug was also tested for patients in stage 1 T1D (^101^) participants received abatacept and 111 placebo). There was higher C-peptide responses to oral glucose tolerance tests with abatacept than placebo, but the treatment did not delay progression to dysglycemia in stage 1 patients with T1D (^64^).

Hydroxychloroquine was tested for patients with stage 1 T1D (n = 273), to prevent or delay the progression to stage 2 T1D. There were no adverse safety concerns in the hydroxychloroquine arm, but the drug did not delay progression to stage 2 T1D (^65^).

A few interventions have been tested for the primary prevention of T1D in patients with a high genetic risk but without autoantibodies. The Trial to Reduce IDDM in Genetically at Risk (TRIGR) Study was a dietary randomized controlled trial designed to determine whether weaning to an extensively hydrolyzed formula in infancy would decrease the risk of type 1 diabetes later in childhood (^66^). The open randomized controlled BABYDIET study investigated the impact of delayed gluten exposure during the first year of life on the development of autoantibodies and T1D (^67^). The Finnish Dietary Intervention Trial for the Prevention of Type 1 Diabetes (FINDIA) was a randomized double-blind clinical trial aimed at determining whether removing bovine insulin from infant formula milk would reduce the incidence of beta-cell autoimmunity. None of these interventions were successful (^68^). The costs of primary prevention trials are very high, and interventions should be harmless, as the study populations include babies who might not develop autoantibodies or T1D throughout their lives (^69^). Therefore, intervention at this point is quite challenging.

#### Antigen-specific based therapies

Antigen-specific therapies offer the possibility of immunologic intervention with minimal toxicity. The use of insulin has been tested to delay or prevent stage 3 T1D in nondiabetic relatives of patients who were at risk for the disease. Patients that had five-year risk of more than 50 percent were randomly assigned to observation or an intervention with low-dose subcutaneous insulin. The incidence of stage 3 T1D was similar in both groups (^70^). For relatives with five-year risk of 26%-50%, oral insulin was tested as intervention (n = 388). Although the rates of progression to stage 3 T1D were similar in both groups, in those with insulin autoantibody (IAA) levels ≥ 80 nU/mL (*n* = 263), there was a trend suggesting benefit in the annual risk of progression to clinical disease (^71^).

Vaccination with subcutaneous application of a recombinant GAD molecule (^20^) mcg GAD-alum) was tested both in individuals with classic T1D aged 10 to 20 years, and in patients with Latent Autoimmune Diabetes of Adults (LADA). There was temporary benefit only in LADA (^72^). A pilot study with the administration of GAD-alum in the inguinal lymph node and VIT D (2,000 IU/day) in 12 newly diagnosed patients with stage 3 T1D led to the maintenance of C-peptide, improved glycemic control and reduction in insulin dose after 15 months (^73^). In a larger study with 109 patients using the same protocol, patients with HLA DR3DQ2 exhibited lower glycemic variability, greater number of patients in partial remission and better pancreatic β cell function (^74^). This vaccine was tested in children with pre-clinical T1D, but it did not change the progression to stage 3 T1D (^75^).

These data suggest that antigen-specific therapies have a modest impact on T1D progression. However, they may be well-suited for patients with specific characteristics such as high specific antibody titers.

#### Additional remarks

The data presented above suggest that multiple non-cellular immunomodulatory therapies could play a significant role in preventing the progression to stage 3 T1D and mitigating β-cell loss, once this stage has begun. While some therapies act by potentializing T regulatory response, others appear to have a direct role in the β cells, the antigen-presenting process, or additional mechanisms that have not been fully understood yet. Although antigen-specific therapies have shown only modest results, they might be suitable for a fraction of patients with specific characteristics or endotypes, alone or in combination with other drugs.

It remains unclear when is the optimal time to initiate immunomodulatory treatment, how to select the appropriate medication, and what outcomes of combination therapies – using other non-specific drugs or antigen-specific agents – might be. Combining drugs with different mechanisms of action seems more appealing due to the multiple pathways involved in T1D development.

Early immunomodulation starting in pre-clinical stages appears more promising, but safety data for the long-term use of non-specific immunomodulatory agents in T1D, especially in children and adolescents, is still lacking. In addition, biomarkers that could identify the ideal population and the ideal moment to intervene for each drug are also a matter of interest in the pursuit of stage 3 T1D prevention.

Furthermore, considering the results of clinical trials and the loss of benefits over time, it could be necessary to implement multiple repeated interventions over time. However, it is crucial to identify treatments with acceptable safety profiles for this strategy.

### Cell therapies for T1D

Cell-based therapies may act as immunoregulatory agents, which would be suitable only for patients with recent-onset stage 3 disease and residual ß cell mass, or to replace β-cells, for patients with any duration of the disease. Immunomodulation may be achieved with Tregs, tolerogenic DCs (tolDCs), hematopoietic stem cells (HSCs) and mesenchymal stem cells (MSCs). They offer the possibility to suppress autoimmunity against islet peptides, while protecting the residual β cell mass that is still present in the early stages of T1D. These therapies have not been tested in individuals in preclinical stages of T1D (stages 1 and 2).

#### Cellular immunomodulation to block the autoimmune attack

##### Regulatory T cells (Tregs) therapy

Tregs are one of the main regulators of immune tolerance (unresponsiveness toward self-tissues) by inhibiting autoreactive T lymphocytes. Functional defects of Treg cells have been reported in T1D (^76,77^). Adoptive transfer of Treg cells in patients with T1D could restore immune tolerance and increase the number of Tregs in circulation.

Phase I clinical studies showed that autologous polyclonal Treg adoptive immunotherapy is feasible and safe both for children and adults with new-onset stage 3 T1D (^78,79^), without infusion reactions or infectious complications. Autologous polyclonal Tregs were successfully isolated from peripheral blood of patients with T1D, expanded ex-vivo, and reinfused into donors. The number of infused cells diminished over time, but some persisted in the circulation for one year or more without acquiring pathological phenotypes. Higher C-peptide levels and lower insulin requirements were reported in the treatment group as compared to others. Further follow-up revealed that the initial therapeutic effects of exogenous Tregs are not sustained over time (^80^). A supplemental administration with a second dose of polyclonal Tregs 6 to 9 months after the first dose resulted in better metabolic outcomes at 12 months of follow-up when compared to those that received none or one dose of exogenous Tregs. However, these benefits were lower than those observed after the first Tregs infusion (^81^).

Despite the substantial benefits of the adoptive transfer of Tregs, it remains to be elucidated if it can lead to immune tolerance and prevention of T1D progression. Two phase II studies on umbilical cord derived-Treg therapy in T1D (ClinicalTrials.gov identifier: NCT02932826 (^82^) and NCT03011021 (^83^) are currently recruiting participants and will contribute to elucidate this. A major challenge for the clinical use of autologous Tregs for T1D is their progressive decline in both number and function over the time (^84^).

Finally, recent efforts on Treg cell-based therapy aim to maximize organ-target homing of infused Tregs and their retention over time (^85^) with therapies that exhibit pancreatic β-cells antigen specificity, for example (^86^). Genetic engineering technologies to generate Treg cells that express a synthetic chimeric antigen receptor (CAR) or a T-cell receptor (TCR) that recognize an islet-specific antigen are also under investigation (^87^).

##### Tolerogenic DC (tolDCs) therapy

DC are cells with a major role in the antigen-presenting process as well as in T cell differentiation and migration, possibly having both pro-inflammatory and anti-inflammatory phenotypes. TolDCs are critical players in regulating immune tolerance. *In vitro*, they exhibit low T cells costimulation capacity, induce anergy of self-reactive T cells through increased provision of inhibitory signals, mediate T cells apoptosis and induce peripheral Tregs and Bregs (regulatory B cells) (^88,89,90^). These tolerogenic properties could contribute to prevent in vivo anti-self-immune responses.

Human tolDCs can be manufactured by exposing human peripheral blood monocytes to differentiation factors, such as granulocyte-macrophage colony-stimulating factor (GM-CSF) and IL-4, that induce cell differentiation towards immature DCs. They are tolerized with anti-inflammatory cytokines (IL-10 and/or TGF-β) or pharmacological agents (dexamethasone, rapamycin, and vitamin D3) (^88,89,90^).

The first clinical trial with tolDCs in T1D patients reduced the expression of costimulatory molecules (CD40, CD80, and CD86) and induced a tolerogenic immunosuppressive phenotype (^91^). A phase II trial is currently ongoing to evaluate the efficacy of these tolDCs in newly diagnosed stage 3 T1D patients (Clinicaltrials.gov ID: NCT023544911) (^92^). Curiously, a phase I placebo-controlled trial with toIDC pulsed with the proinsulin peptide C19-A3 in patients with long-standing T1D showed safety, slight decrease in HbA1c (average 0.34%), reduced antigen-specific T cell proliferation and cytokine response to the vaccine peptide for more than two years (^93^), without changes in general immune competence (^94^). A phase I/II clinical trial is ongoing to evaluate the safety and tolerability of autologous tolDCs primed with peptides and differentiated with mesenchymal stem cells (Clinicaltrials.gov ID: NCT05207995) (^95^) aiming to halt or delay the autoimmune destruction process in T1D.

##### Hematopoietic Stem Cells (HSC) therapy

HSCs transplantation has been investigated as a strategy to induce tolerance in patients with T1D. Autologous nonmyeloablative HSC transplantation was performed in 23 patients with newly diagnosed stage 3 T1D that received high-dose cyclophosphamide plus ATG as a conditioning (immune ablative) regimen. Transplantation increased pancreatic β cells function and induced prolonged absence or reduction of daily insulin doses in the majority of the patients, with acceptable incidence of adverse effects (^96,97^).

The rationale for HSCs therapy in autoimmune diseases is the restoration of immune tolerance through (I) deletion of autoreactive immunologic memory of patients using immunosuppressants (condition regimen), and (II) re-infusion of previously collected HSC which are cells without immunologic memory. This aims to restore self-tolerance and to achieve a profound reconfiguration of the immune system (immune “resetting”), without long-term immunosuppression, to preserve residual β-cell mass (^98^).

Independence from exogenous insulin, lower insulin dose requirement, decreased HbA1c levels, and improvement of C-peptide levels were reported after autologous HSCs therapy in patients with T1D (^96,97,99,100,101,102^). Immunoablative conditioning may account for some of these benefits. Still, patients that underwent intervention were free from macrovascular and microvascular diabetic complications after a mean follow-up of 8 years, while 21.5% and 13.8% of placebo- treated group experienced microvascular complications and nephropathy, respectively (^103^).

Despite all these favorable outcomes, the effectiveness of autologous HSCs therapy in T1D seems to be time-limited and a significant proportion of patients return to insulin treatment. Studies reported a lack of persistent remission following initial improvement in patients with T1D treated with autologous HSCs therapy (^102,104^). In addition, the effectiveness of HSCs therapy and duration of the remission period vary according to the immune status of T1D patients. Individuals with less islet-specific autoreactive CD8+ T cells at baseline experienced prolonged remission and presented greater C-peptide levels after therapy than those with lower frequencies of these cells (105. D’Addio and cols. (^100^) reported that treatment within 6 weeks after diagnosis resulted in higher rates of insulin independency (^82^)% *vs.* 40%) However, this was not confirmed by Snarski and cols. (^105^). The requirement of a conditioning regimen is also a barrier for the use of HSC as a treatment for T1D in clinical practice as it can lead to a wide range of complications that can result in death. The risks associated with HSCs therapy and the risks of diabetes-associated complications must be weighed to define the best intervention option to patients (^106^).

##### Mesenchymal stem/stromal cells

Mesenchymal stem/stromal cells (MSCs) are multipotent cells with secretory regenerative capacity. Their secretory profile includes cytokines and growth factors with anti-inflammatory and immune-modulatory capacities, making this source of adult stem cells valuable for autoimmune diseases (^107^). MSCs have been widely investigated in thousands of clinical trials (^108,109,110^) and are promising as cell-based products in regenerative medicine.

MSCs can be isolated from different adult tissues. The most frequent sources that have been tested in clinical trials are bone marrow, adipose tissue, and umbilical cord (^111,112^) mainly due to the ease of tissue harvesting. MSCs derived from bone marrow, adipose tissue, and umbilical cord revealed subtle molecular and phenotypic differences which do not necessarily result in differences in results obtained in clinical trials for T1D (^113,114^).

The pioneering clinical trials with MSC-based therapy for T1D were based in autologous sources mainly administered by intravenous infusion (^111^), but allogeneic MSC source was also investigated (^115,116^).

An allogeneic MSC source is particularly interest, since autoimmune diseases can compromise the anti-inflammatory and immune-modulatory capacities of MSCs (^117^), although one pre-clinical study showed that MSCs isolated from the bone marrow of newly diagnosed T1D patients revealed similar clinical results compared with MSCs from healthy individuals (^118^). More importantly, MSC-based allogeneic therapies may reduce costs and increase reproducibility of these regenerative therapies, since cells from only one donor could be isolated and cryopreserved for long-term in biobanks to be used for at least dozens of patients. This would reduce the need to harvest and isolate MSCs for each medical intervention and alleviate concerns regarding donor MSC variability.

MSC-based therapies are safe, since no clinical trial reported serious adverse effects, and also seems to be effective. Studies have reported slight improvement in several clinical parameters of diabetes such as C-peptide and HbA1c, suggesting a preservation of pancreatic β cell function. However, clinical improvement is not maintained in the long term (^111,116^), leading to the hypothesis that repeated injections of MSCs could improve their regenerative effect on the preservation of pancreatic β-cell function in T1D (^117^). Our group has demonstrated that allogenic adipose tissue-derived stromal/stem cells (ASCs) transplantation combined with VITD without immunosuppression was safe and associated with lower insulin requirements, better glycemic control, and transient better pancreatic function in recent onset stage 3 T1D, but the potential benefits do not seem to be sustained (^115,116^). Peripancreatic infusion of stem cells is also under investigation in pre-clinical trials, aiming to optimize their regenerative capacity, since blood systemic injection leads to a massive loss of cells in other organs non-related to T1D. A recent study showed that transplant of microencapsulated MSCs into the peritoneal cavity of nonobese diabetic mice induced sustained remission of T1D (^118^).

#### Cellular therapies for β-cell replacement

Since T1D is characterized by pancreatic β cell destruction, its replacement with allogeneic islet cell transplantation or cells derived from pluripotent stem cells holds the potential to cure the disease.

Allogeneic islet transplantation has progressed immensely over the last decades. Although there is evidence that 73% of patients that underwent the intervention required re-infusion and only 8% remained insulin independent after 20 years, this procedure has shown to be safe and well-suited for patients with brittle diabetes and severe hypoglycemia. Database from the Collaborative Islet Transplant Registry (CITR) shows that most patients (87.5% in 1 year, 71% in 2 years) that received an allogenic islet transplantation achieved stable glycemic control (HbA1c < 7% without severe hypoglycemia). While the risks of life-long immunosuppression seem to be too high for patients with T1D in general, they are worthy for adults that have not achieved target HbA1c due to repeated episodes of severe hypoglycemia, despite adequate care (^119,120,121^).

Donor shortage and immunosuppressant-related complications are major hurdles for the wide use of islet transplantation for T1D treatment. Long-term immunosuppression may be associated with a decline in glomerular filtration rate, increase the risk of infections and tumorigenesis. The survival, proliferation, and functionality of isolated islets following transplantation are issues that still need to be addressed. A loss of up to half of grafted cells is expected in the first days after transplantation, especially due to delayed graft revascularization. Therefore, other cell therapies and alternatives to protect the transplanted cells are under investigation (^122,123,124^).

Xenogeneic islets and stem cell-differentiated β cells emerged as potential alternatives. In theory, they could offer unlimited source of islets to overcome the donor shortage problem (^125^). The most studied xenogeneic islets are derived from pigs. The ability to produce a large number of islets and an insulin that is similar to that of humans makes this type of therapy attractive. However, safety is an important concern in xenotransplantation. In pigs, porcine endogenous retroviruses (PERVs) are well known pathogens integrated through pig genome. Other potential sources of zoonosis such as herpes virus and cytomegalovirus should also be considered. Matsumoto and cols. transplanted microencapsulated pig islets in eight patients with T1D without immunosuppressant coverage and demonstrated long-term improvement of hyperglycemia and restoration of impaired hypoglycemia awareness (^126^). The precise edition of the genome to silence genes (CRISPR/Cas technology) responsible for allogeneic immune response started to be tested in animal models and could support the xenotransplantation of islets in clinical practice (^127^).

Human pluripotent stem cells (hPSCs) represents another alternative source for β cell replacement. hPSCs can be derived from the inner cell mass of the embryo known as embryonic stem cells (ESCs) and from reprogramming adult somatic cells known as induced pluripotent stem cells (iPSCs). Since ESCs are derived from human embryos, their transplantation has the potential of allograft rejection and also raises ethical concerns that could be avoided by the use of iPSCs (^128,129,130,131^).

HPSCs can be expanded in vitro to provide an unlimited source of β cells and have the ability to differentiate into pancreatic progenitors that mature in vivo into glucose-responsive β cells after transplantation. Alternatively, these cells can be differentiated into pancreatic β cells in vitro and then transplanted to patients. The variability in the differentiation efficiencies of different hPSCs lines remains a challenge (^129,130^) The differentiation of hPSCs into pancreatic β cells has achieved substantial progress (^129,130,131^). Efforts have been made to isolate and transplant a homogeneous and pure population of cells, avoiding the selection of teratogenic cells and the risk of tumorigenesis (^132^).

The recipient’s immune response to donor islets is another important barrier of islet transplantation derived from ESCs. The current immunosuppressive protocol to improve the outcome of islet transplantation recommends depletion of T-cells, TNF-α inhibitors during induction of immune suppression and use of a calcineurin inhibitor or mTOR inhibitors for maintenance. This approach increases the risk of impaired renal function and infections. To overcome these complications, some options have been developed such as the induction of immune tolerance, physical barriers, simultaneous infusion of Tregs and immune-evasive insulin-producing cells (^123,124,130,133^). There are successful preclinical reports of co-transplantation of bone marrow-derived HSCs (^123,131,132^) or MSCs (^134,135,136^) with allogeneic islets inducing immune tolerance, but clinical data are still lacking.

Micro-and macrocapsules with the ability of blocking entrance of host immune cells can act as physical barriers. Microcapsules usually encapsulate one islet in one capsule. Several phase 1/2 clinical trials evaluated the safety and efficacy of alginate microencapsulation in humans with promising results. However, there are reports of inconsistent results regarding the biocompatibility and immunogenicity of alginate capsules. Efforts have been made to enhance the capsules (^134,137^).

Macrocapsules encapsulate a large number of islets in chamber-like devices and are composed by polymers. While microencapsulation devices allow for easier nutrient and oxygen exchange, macroencapsulation devices are safer with easy monitoring and retrieval (^130,132,133^,,^137^).

Currently, we have proof of concept of the safety and potential efficacy of β cells generated from hPSCs using two different approaches: subcutaneous implantation of immature progenitor cells in a microencapsulation device; or intraportal delivery of in vitro matured, glucose-responsive cells. Encapsulated pancreatic endodermal cells, differentiated from human ESCs, have been tested in some clinical trials (NCT03162926, NCT03163511, NCT02239354 and NCT02939118) with promising results in terms of cell survival and maturation. Subcutaneous implants were well tolerated with minor adverse effects, but the engraftment and insulin production were challenging (138.

A phase 1/2 trial (VX-880, NCT04786262) transplanted ESC differentiated β-like cells to 17 patients with T1D with previous history of severe hypoglycemia and hypoglycemic unawareness. The unencapsulated cells were delivered by the portal vein combined with immunosuppressant use. The patients experienced an increase in fasting and postprandial C-peptide, a decreased HbA1c and reduced insulin requirement (^139^). Insulin independence was described in a few patients. Long-term data with larger numbers of patients are still awaited. The trial has been temporarily suspended due to two deaths, but an independent investigation indicated that neither of them were related to the treatment and the study was resumed.

Over the past two decades, major advances allowed the generation of stem cell-derived β-like cells that share many features with genuine cells. However, producing fully functional mature cells remains challenging. Further studies are required to validate the safety and efficacy of the current promising options. Finally, the cost-effectiveness of β cell replacement therapy is a crucial factor for its clinical translation and scalability (^130,131,132^). Strategies such as immune modulation of hPSCs by deletion of HLA antigens, immune-cloaking and incorporation of suicide genes could produce universal donor cell lines that would allow wide-scale clinical application of cell therapy for the treatment of T1D (^122,123,129,132,134,137^). The combination of iPSCs with gene-editing technologies seems to be a promising tool for future precision medicine.

## FUTURE DIRECTIONS

Subcutaneous insulin therapy has been the mainstay of T1D treatment for more than 100 years, to compensate for the lack of endogenous secretion. Although most autoimmune diseases are treated with immunomodulatory agents, this is still not a reality for autoimmune diabetes. Recently, FDA has approved the first disease-modifying therapy for T1D (teplizumab) and other therapies have shown promising results. Substantial advances are expected in this field soon. Therefore, it is likely that, in the forthcoming century, we will not only offer insulin therapy but also disease-modifying therapies, to interrupt the autoimmune attack and/or replace lost ß cells. As T1D has a multifactorial origin, combination therapies including agents with different synergistic mechanisms of action seem appealing, exploring interactions between the immune system and other locations, such as the gut. These combined therapies may have an impact on diabetes prevention and progression.

Some major challenges must be faced to establish this new reality, such as identifying candidates within the ideal moment to receive intervention, which depends on cost-effective screening programs for T1D, and establishing therapies with acceptable adverse events risks and realistic costs. With the advancement of tissue engineering approaches, cell-based therapies aiming not only to achieve immunomodulation but also ß cell replacement will be pursued and improved, leading to more effective and long-lasting treatments for T1D.
